# Analysis of the risk difference in post-spinal anesthesia hypotension between primiparas and multiparas in cesarean section

**DOI:** 10.3389/fsurg.2025.1617342

**Published:** 2025-06-18

**Authors:** Jizheng Zhang, Jinli Che, Xiaohua Sun, Yi Li, Wanlu Ren

**Affiliations:** Gynaecology and Obstetrics Department, Tianjin Hospital Affiliated to Tianjin University, Tianjin, China

**Keywords:** cesarean section, hypotension, interaction, multiparas, primiparas, spinal anesthesia

## Abstract

**Introduction:**

This study aimed to investigate the impact of maternal type and its interactions on the incidence of hypotension following spinal anesthesia.

**Methods:**

In this retrospective cohort study, both primiparous and multiparous women were included. Demographic, pregnancy-related, and hemodynamic data were collected. Univariate and multivariate logistic regression analyses were performed to evaluate the association between these factors and the occurrence of hypotension after spinal anesthesia. Additionally, multivariate models with and without maternal type were constructed, followed by interaction analysis.

**Results:**

Primiparous women had a lower median age and slightly greater weight gain during pregnancy compared to multiparous women. They also exhibited significantly higher pleth variability index (PVI) and heart rate (HR), while heart rate variability (HRV) was significantly lower. Univariate regression analysis identified maternal type, age, weight gain during pregnancy, estimated fetal weight, PVI, HR, HRV, and systolic blood pressure (SBP) as significant predictors of hypotension. Multivariate model analysis showed that adding the variable of parity significantly improved the model’s ability to discriminate the occurrence of hypotension (Model 2 AUC = 0.815 vs. Model 1 AUC = 0.740). Interaction analysis revealed significant interactions between heart rate variability (HRV), systolic blood pressure (SBP), gestational weight gain, and parity, suggesting that these physiological characteristics are more strongly associated with hypotension in primiparas.

**Discussion:**

In conclusion, primiparous women are at significantly higher risk of developing hypotension after spinal anesthesia than multiparous women. Baseline perfusion index (PI), estimated fetal weight, and baseline PVI are key contributing factors to this outcome.

## 1 Introduction

Spinal anesthesia and general anesthesia are commonly used anesthesia methods in laparoscopic surgeries or cesarean sections (1). Spinal anesthesia, with its rapid onset, ease of administration, and minimal impact on mother and infant, has become the preferred anesthesia method for cesarean sections. In contrast, general anesthesia, although suitable for certain special cases, may cause greater systemic effects. Hypotension following spinal anesthesia is one of the most common complications during cesarean section and typically presents as a sudden and marked drop in blood pressure, especially within a short period after anesthesia administration (2–4). T This condition not only disrupts the hemodynamic stability of the mother but may also result in a range of serious adverse outcomes for both the mother and the neonate (5). For the mother, an abrupt decline in blood pressure can lead to insufficient perfusion of vital organs such as the heart, brain, and kidneys, potentially causing acute organ dysfunction and, in severe cases, life-threatening complications (6). For the fetus, maternal hypotension can reduce uteroplacental blood flow, compromising oxygen and nutrient delivery to the placenta, thereby increasing the risk of neonatal asphyxia, low Apgar scores, and hypoxic-ischemic encephalopathy (7). Furthermore, maternal hypotension may prolong the delivery process and raise the incidence of postoperative complications, ultimately impacting the overall recovery during the postpartum period. Therefore, identifying and preventing hypotension after spinal anesthesia is of critical clinical importance for safeguarding maternal and neonatal health and improving postpartum outcomes.

At present, studies have explored the effects of various factors on hypotension after spinal anesthesia, such as some hemodynamic parameters, pleth variability iindex (PVI) (8), heart rate variability (HRV), etc (9–11). Due to the first changes in various bodily systems experienced during pregnancy, primiparous women may face more physiological challenges, such as differences in heart rate, blood perfusion variability, and weight changes compared to multiparous women. These changes may affect vascular reactivity and the metabolism of anesthetic drugs. However, whether these known factors related to hypotension (such as PVI, heart rate, and heart rate variability) have different degrees of association in primiparas and multiparas remains to be further studied.

The aim of this study is to investigate the differences in the incidence of hypotension following spinal anesthesia between primiparous and multiparous women. By constructing univariate and multivariate regression models, as well as conducting interaction analyses, this study aims to explore whether key factors associated with the occurrence of hypotension exhibit different strengths of association in primiparas compared to multiparas. The ultimate goal is to provide more accurate risk assessment tools to support clinical decision-making in anesthesia management.

## 2 Materials and methods

### 2.1 Patients

The study included parturients who underwent cesarean section with spinal anesthesia at our hospital, categorized into primiparas and multiparas based on maternal type. The study period was from May 2021–May 2024. Inclusion criteria were: parturients undergoing cesarean section; eligibility for spinal anesthesia prior to surgery; singleton full-term pregnancy; complete clinical data including hemodynamic indicators and relevant clinical information. Exclusion criteria were: Severe cardiovascular disease, including NYHA class III or above heart failure, recent (within 6 months) myocardial infarction, or arrhythmias requiring long-term anti-arrhythmic medication or device implantation; Hepatic insufficiency, defined as ALT or AST exceeding twice the upper limit of normal, or confirmed diagnosis of liver cirrhosis by imaging or pathology; Renal insufficiency, defined as eGFR <60 ml/min/1.73 m^2^; Other systemic diseases such as systemic lupus erythematosus, malignant tumors, or psychiatric disorders that affect compliance; Conversion from spinal anesthesia to general anesthesia during surgery; Contraindications to or inability to tolerate spinal anesthesia during pregnancy. A total of 525 parturients were initially screened. Among them, 8 were excluded due to severe cardiovascular diseases (e.g., heart failure, arrhythmias); 11 due to hepatic or renal insufficiency; 6 due to intraoperative conversion to general anesthesia; 47 due to twin or preterm pregnancies; and 33 due to missing key clinical data. Ultimately, 420 parturients were included in the final analysis. We conducted a power analysis to ensure that the study has sufficient statistical power to detect clinically meaningful differences. Using a medium effect size, a power of 0.8, and a significance level of 0.05 as criteria, the required sample size was calculated. For categorical data, each group needs at least 93 participants, while for continuous data, each group requires at least 64 participants.

### 2.2 Anesthesia method

All parturients were positioned in the left lateral position. After successful subarachnoid puncture with cerebrospinal fluid flow observed, 1.5 ml of 0.75% ropivacaine hydrochloride was injected. Immediately after the injection, the patient was placed in a supine position with a 15-degree left tilt. After spinal anesthesia, a cold stimulus test (alcohol swab) was used to assess the sensory block height, ensuring it reached T6-T4. During the surgery, oxygen was continuously administered at a flow rate of 5 l/min. All parturients were monitored according to a standardized protocol, with blood pressure and heart rate recorded every 1 min after spinal anesthesia until 10 min after fetal delivery, and then every 3–5 min thereafter, ensuring consistency in the frequency and method of vital sign monitoring. Hypotension was defined as a systolic blood pressure of less than 90 mmHg or a decrease of more than 20% from baseline systolic blood pressure within 30 min after spinal anesthesia. During this period, all parturients adopted a 15-degree left lateral tilt position to reduce the impact of aortocaval compression. All parturients received a rapid preload of 500 ml lactated Ringer’s solution prior to the initiation of spinal anesthesia. Simultaneously, an additional 500–1,000 ml of lactated Ringer’s solution was administered as co-loading during the local anesthetic injection to reduce the risk of hypotension caused by sympathetic blockade. In cases of hypotension, standardized management protocols were implemented. These included positioning the parturient in a 15-degree left lateral tilt, rapid infusion of lactated Ringer’s solution or balanced salt solution (500–1,000 ml), and administration of vasopressors. Ephedrine (5–10 mg) was used as the first-line agent and additional doses were administered based on the blood pressure response. Vital signs were continuously monitored, oxygen was provided if necessary, and all interventions were thoroughly documented.

### 2.3 Data collection

The basic information includes age, BMI before pregnancy, gestational age, weight gain during pregnancy, and whether there is hypertension history, diabetes history, cardiovascular disease history, kidney disease history, pregnancy diabetes and other diseases. Regarding the factors of anesthesia operation, record the puncture sites (L2–L3, L3–L4, L4–L5). The baseline hemodynamic indicators, including perfusion index (PI), pleth variability index (PVI), heart rate (HR), and heart rate variability (HRV, represented by the LF/HF ratio), were measured before spinal anesthesia while the parturients were in a calm and resting state to ensure a true and stable preoperative baseline. In addition, baseline blood pressure parameters such as systolic blood pressure (SBP) and diastolic blood pressure (DBP) were also included in the analysis to assess the risk of hypotension after spinal anesthesia. These indicators are measured in a supine position before anesthesia.

### 2.4 Statistical analysis

Continuous variables were expressed as median (minimum–maximum) and compared using either the t-test or the Wilcoxon rank-sum test, as appropriate. Categorical variables were presented as frequency (percentage) and analyzed using the chi-square test. Maternal type was coded as 1 for primiparous women and 0 for multiparous women, and treated as an independent variable to assess its association with hypotension following spinal anesthesia using univariate logistic regression analysis. To further identify risk and protective factors significantly associated with the occurrence of hypotension, multivariable logistic regression analysis was conducted. Two models were constructed: Model 1 (excluding maternal type) and Model 2 (including maternal type). The discriminatory ability of maternal type in identifying the risk of hypotension was evaluated by comparing the area under the receiver operating characteristic curve (AUC) and the Hosmer–Lemeshow goodness-of-fit test results between the two models. To facilitate the interpretation of interaction results and the identification of high-risk groups, we dichotomized continuous risk and protective factors at their median values. Values below the median were coded as 0 and those above as 1. Interaction analyses between maternal type and each risk or protective factor were conducted to explore potential synergistic or antagonistic effects on the occurrence of hypotension after spinal anesthesia.

## 3 Results

### 3.1 Differences in demographics, pregnancy information, and hemodynamic indicators between primiparous and multiparous women

The results showed that the median age of primiparous women was 28 years (22–37 years), while that of multiparous women was 33 years (22–41 years). The significant difference between the two (*P* = 0.00618) indicates that the age of multiparous women is generally higher. The median weight gain during pregnancy for primiparous women is 10.3 kg, while for multiparous women it is 10.0 kg. The difference between the two groups was small, but the weight gain of primiparous women was slightly higher (*P* = 0.0285). The median PVI value for primiparous women is 16.4 (6.1–23.4%), while for multiparous women it is 13.3 (6.1–23.5%). The significant difference in PVI (*P* = 4.14 × 10^−5^) indicates a high variability in pulse perfusion among primiparous women. The median HR for primiparous women was 87 beats per minute (66–103 beats per minute), while for multiparous women it was 81 beats per minute (66–102 beats per minute), with a significant difference (*P* = 0.00385). The median HRV (LF/HF ratio) of primiparous women was 1.7 (1.2–2.6), while that of multiparous women was 2.0 (1.2–2.6). The difference in this ratio between the two groups was significant (*P* = 0.00149), and multiparous women had stronger sympathetic nervous activity. Other factors, such as hypertension history, diabetes history, gestational diabetes, PI, SBP, and DBP, were not significant between the two groups (Table 1).

**Table 1:** Demographic, pregnancy-related, and hemodynamic differences between primiparas and multiparas.

Variables	All Patients (*n* = 420)	Primipara (*n* = 210)	Multipara (*n* = 210)	*p*-value
Age (years)	30 (22–41)	28 (22–37)	33 (22–41)	0.00618
Weight gain during pregnancy (kg)	10.2 (7.8–12.3)	10.3 (7.8–12.3)	10.0 (7.8–12.2)	0.0285
Pre-pregnancy BMI				0.065611
Underweight (BMI < 18.5)	27 (6.43%)	17 (8.1%)	10 (4.76%)	
Normal weight (18.5 ≤ BMI < 24.9)	303 (72.14%)	141 (67.14%)	162 (77.14%)	
Overweight or Obesity (BMI ≥ 25)	90 (21.43%)	52 (24.76%)	38 (18.1%)	
Hypertension history				0.091596
Yes	48 (11.43%)	18 (8.57%)	30 (14.29%)	
No	372 (88.57%)	192 (91.43%)	180 (85.71%)	
Diabetes history				0.248192
Yes	29 (6.9%)	18 (8.57%)	11 (5.24%)	
No	391 (93.1%)	192 (91.43%)	199 (94.76%)	
Cardiovascular disease history				0.364783
Yes	72 (17.14%)	32 (15.24%)	40 (19.05%)	
No	348 (82.86%)	178 (84.76%)	170 (80.95%)	
Kidney disease history				0.074286
Yes	8 (1.9%)	7 (3.33%)	1 (0.48%)	
No	412 (98.1%)	203 (96.67%)	209 (99.52%)	
Gestational Diabetes Mellitus				0.198158
Yes	23 (5.48%)	15 (7.14%)	8 (3.81%)	
No	397 (94.52%)	195 (92.86%)	202 (96.19%)	
Fetal weight estimation (g)	3429 (2519–4311)	3464 (2519–4308)	3404 (2534–4311)	0.854
Gestational age (week)				0.721277
37–38	90 (21.43%)	47 (22.38%)	43 (20.48%)	
39 or more	330 (78.57%)	163 (77.62%)	167 (79.52%)	
Puncture site				0.08762
L2–L3	18 (4.29%)	8 (3.81%)	10 (4.76%)	
L3–L4	352 (83.81%)	184 (87.62%)	168 (80%)	
L4–L5	50 (11.9%)	18 (8.57%)	32 (15.24%)	
Perfusion Index, PI	7.1 (1.1–13.5)	6.6 (1.1–13.5)	7.6 (1.1–13.4)	0.32
Pulse Perfusion Variability Index, PVI (%)	15.0 (6.1–23.5)	16.4 (6.1–23.4)	13.3 (6.1–23.5)	4.14 × 10^−5^
Heart Rate, HR (bpm)	84 (66–103)	87 (66–103)	81 (66–102)	0.00385
Heart Rate Variability, HRV (LF/HF ratio)	1.9 (1.2–2.6)	1.7 (1.2–2.6)	2.0 (1.2–2.6)	0.00149
Systolic Blood Pressure, SBP (mmHg)	101.9 (90.3–109.5)	102.8 (90.3–109.4)	101.2 (90.4–109.5)	0.658
Diastolic Blood Pressure, DBP (mmHg)	74.8 (62.0–87.9)	75.2 (62.0–87.8)	74.4 (62.1–87.9)	0.636

### 3.2 Univariate logistic regression analysis of factors affecting the occurrence of hypotension after spinal anesthesia

Firstly, the type of parturient (primiparous vs. multiparous) significantly affects the occurrence of hypotension, with primiparous women having a higher risk of hypotension (B = 1.735, *P* = 0.000). In addition, age (B = 0.051, *P* = 0.005), changes in BMI (B = 0.201, *P* = 0.010), estimated fetal weight (B = 0.001, *P* = 0.001), perfusion index (PI) (B = 0.095, *P* = 0.001), pleth variability index index (PVI) (B = 0.064, *P* = 0.002), heart rate (HR) (B = 0.023, *P* = 0.019), heart rate variability (HRV) (B = 0.624, *P* = 0.001), and systolic blood pressure (SBP) (B = −0.045, *P* = 0.002) are all significantly correlated with the occurrence of hypotension. Specifically, increasing age, higher weight gain during pregnancy, higher estimated fetal weight, higher PI and PVI, higher HR and HRV, and lower SBP are all associated with an increased risk of hypotension (Table 2).

**Table 2:** Univariate logistic regression analysis of factors influencing hypotension after spinal anesthesia.

Variables	B	Std Error	Z-Value	*p*-value
Puncture site (L2–L3)	0.742	0.485	1.528	0.126
Puncture site (L3–L4)	0.461	0.288	1.601	0.109
Puncture site (L4–L5)	−1.003	0.369	−2.718	0.007
Age	0.051	0.018	2.777	0.005
Weight gain during pregnancy	0.201	0.078	2.565	0.010
Hypertension history	−0.029	0.317	−0.090	0.928
Diabetes history	−0.335	0.415	−0.808	0.419
Cardiovascular disease history	0.318	0.262	1.216	0.224
Kidney disease history	−0.624	0.823	−0.758	0.448
Gestational Diabetes Mellitus	0.046	0.440	0.105	0.916
Fetal weight estimation	0.001	0.000	3.211	0.001
Pre-pregnancy BMI	−0.618	1.159	−0.534	0.594
Gestational age	−0.209	1.229	−0.170	0.865
Perfusion Index, PI	−0.074	0.028	−2.658	0.008
Pulse Perfusion Variability Index, PVI	0.064	0.021	3.121	0.002
Heart Rate, HR	0.023	0.010	2.336	0.019
Heart Rate Variability, HRV (LF/HF ratio)	−0.739	0.253	−2.922	0.003
Systolic Blood Pressure, SBP	−0.045	0.014	−3.120	0.002
Diastolic Blood Pressure, DBP	−0.007	0.014	−0.497	0.619
Maternal Type	1.735	0.224	7.732	0.000

### 3.3 Multivariate logistic regression analysis of the effect of maternal type on hypotension after spinal anesthesia

**Figure 1:**
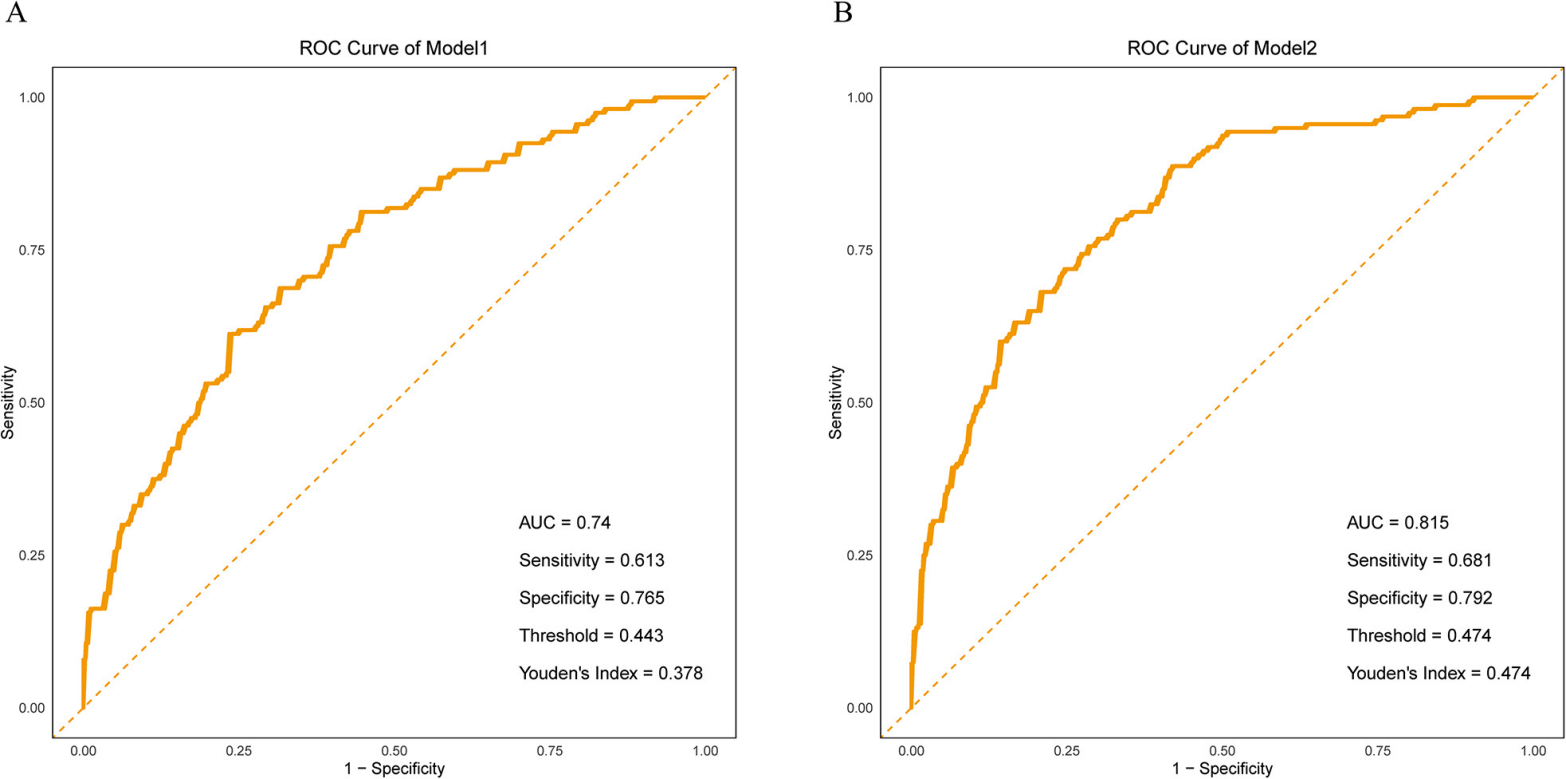
**(A)** ROC curve of model 1. **(B)** ROC curve of Model 2.

The results demonstrated that Model 2, which incorporated maternal type, exhibited improved discriminatory ability for identifying spinal anesthesia-induced hypotension, as indicated by a higher AUC compared to Model 1 (0.815 vs. 0.740) (Figures 1A,B). The Hosmer–Lemeshow test for Model 1 indicated good model fit (*χ*² = 6.10, df = 8, *p* = 0.637), suggesting no significant difference between predicted probabilities and observed outcomes. For Model 2, the Hosmer–Lemeshow test yielded a *χ*² value of 0.945 with 8 degrees of freedom and a *p*-value of 0.999 (*p* > 0.05), indicating an excellent agreement between predicted probabilities and actual observations. Moreover, the higher *p*-value for Model 2 implies a better model fit and stronger agreement between predicted and observed outcomes. These findings highlight the critical role of maternal type (primipara vs. multipara) in the occurrence of hypotension following spinal anesthesia. In addition, the multivariate regression results of Model 1 also showed that perfusion index (PI), fetal weight estimation, heart rate variability (HRV), pleth variability index index (PVI), systolic blood pressure (SBP), age, puncture site (L4–L5), and changes in body mass index (BMI) were significant influencing factors. Specifically, PI, HRV, PVI. The increase in age and weight gain during pregnancy are both associated with an increased risk of hypotension and are risk factors, while higher baseline systolic blood pressure and puncture site (L4–L5) are associated with a reduced risk of hypotension and are protective factors. In Model 2, the type of parturient (primiparous and multiparous) also showed a significant impact (OR = 1.390, *p* = 0.000). In addition, the heart rate change was slightly higher than the critical significance (*p* = 0.060). In summary, multiple physiological indicators and maternal types play independent roles in the occurrence of hypotension after spinal anesthesia (Table 3).

**Table 3:** Multivariate logistic regression analysis of factors influencing hypotension after spinal anesthesia.

Term	B	Std error	Statistic	*p*-value	OR	CI-lower	CI-upper
Model 1							
Perfusion Index, PI	−0.016	0.006	−2.553	0.011	0.984	0.973	0.996
Fetal weight estimation	0.000	0.000	2.943	0.003	1.000	1.000	1.000
Heart Rate Variability, HRV (LF/HF ratio)	−0.173	0.054	−3.187	0.002	0.841	0.756	0.935
Pulse Perfusion Variability Index, PVI	0.011	0.004	2.433	0.015	1.011	1.002	1.020
Systolic Blood Pressure, SBP	−0.009	0.003	−3.019	0.003	0.991	0.985	0.997
Age	0.013	0.004	3.286	0.001	1.013	1.005	1.021
Puncture siteL4 L5	−0.160	0.068	−2.341	0.020	0.853	0.746	0.974
Weight gain during pregnancy	0.049	0.017	2.886	0.004	1.050	1.016	1.085
Heart Rate, HR	0.004	0.002	1.820	0.069	1.004	1.000	1.008
Model 2							
Perfusion Index, PI	−0.012	0.006	−2.174	0.030	0.988	0.977	0.999
Fetal weight estimation	0.000	0.000	3.014	0.003	1.000	1.000	1.000
Heart Rate Variability, HRV (LF/HF ratio)	−0.156	0.050	−3.101	0.002	0.855	0.775	0.944
Pulse Perfusion Variability Index, PVI	0.010	0.004	2.360	0.019	1.010	1.002	1.018
Systolic Blood Pressure, SBP	−0.008	0.003	−2.669	0.008	0.992	0.987	0.998
Age	0.012	0.004	3.320	0.001	1.012	1.005	1.020
Puncture site L4 L5	−0.176	0.064	−2.767	0.006	0.839	0.740	0.950
Weight gain during pregnancy	0.040	0.016	2.538	0.012	1.041	1.009	1.074
Heart Rate, HR	0.004	0.002	1.886	0.060	1.004	1.000	1.008
Maternal Type	0.330	0.042	7.902	0.000	1.390	1.281	1.509

### 3.4 Analysis of the interaction between maternal types, risk factors, and protective factors

**Figure 2:**
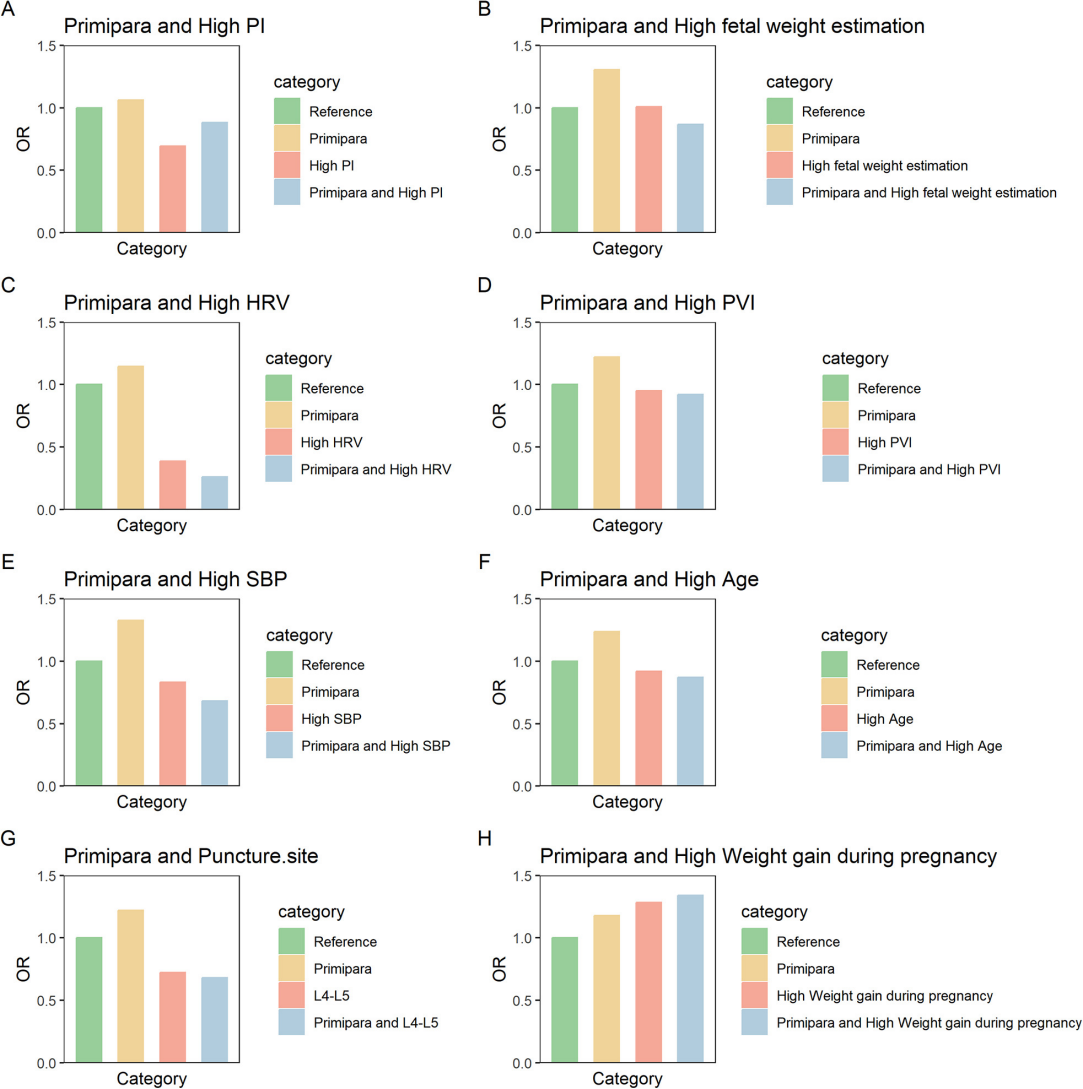
Visual analysis of the odds ratios (OR) for the interaction between maternal type and **(A)** PI **(B)** estimated fetal weight **(C)** HRV **(D)** PVI **(E)** SBP **(F)** age **(G)** puncture site **(H)** gestational weight gain.

We set the risk factors or protective factors as 0, with maternal type 0 (multiparous women) as the reference group. The odds ratios (ORs) and confidence intervals (CIs) for the other groups were adjusted based on the reference group. The results showed a significant interaction between the HRV group and maternal type group (Maternal Type * HRV Group) (*p* < 0.001), with the OR value for the interaction being lower than that for either the maternal type group or the HRV group alone. This suggests that HRV has a protective association, which is further strengthened by maternal type—indicating that the association between HRV and reduced risk of hypotension is more pronounced in primiparas (Figure 2C). The interaction between the SBP group and maternal type group (Maternal Type * SBP Group) was significant (*p* = 0.026), but the OR value was lower than that for either the maternal type group or the SBP group alone. Maternal type enhanced the protective association of baseline SBP, indicating that baseline SBP is more strongly associated with a reduced risk of hypotension in primiparas. A higher baseline SBP significantly reduces the risk of hypotension in primiparas women (Figure 2E). The interaction between weight gain during pregnancy and maternal type (Maternal Type * Weight gain during pregnancy Group) was significant (*p* < 0.001), and the OR value was higher than that for either the weight gain during pregnancy group or the maternal type group alone. There was also a synergistic effect, meaning that the impact of weight gain during pregnancy on hypotension is more pronounced in primiparas women (Figure 2H). No interaction was found between other factors such as age, PI, PVI, and maternal type (Table 4) (Figures 2A,B,D,F,G).

**Table 4:** Interaction analysis of maternal type with risk and protective factors.

Term	B	Std error	Statistic	*p*-value	OR	CI-lower	CI-upper	B adj	OR adj	CI-lower adj	CI-upper adj
Reference	0.253	0.046	5.508	0.000	1.287	1.177	1.408	0.000	1.000	1.000	1.000
Maternal Type	0.313	0.062	5.044	0.000	1.367	1.211	1.544	0.060	1.062	1.029	1.096
PI Group	−0.114	0.062	−1.831	0.068	0.893	0.791	1.008	−0.367	0.693	0.671	0.715
Maternal Type * PI Group	0.127	0.088	1.452	0.147	1.136	0.956	1.349	−0.126	0.882	0.812	0.957
Reference	0.124	0.043	2.854	0.005	1.132	1.040	1.232	0.000	1.000	1.000	1.000
Maternal Type	0.390	0.061	6.365	0.000	1.478	1.310	1.666	0.266	1.305	1.260	1.352
Fetal weight estimation Group	0.133	0.061	2.173	0.030	1.143	1.013	1.289	0.009	1.009	0.974	1.045
Maternal Type * Fetal weight estimation Group	−0.019	0.087	−0.220	0.826	0.981	0.828	1.163	−0.143	0.867	0.795	0.945
Reference	0.179	0.041	4.352	0.000	1.197	1.104	1.297	0.000	1.000	1.000	1.000
Maternal Type	0.315	0.062	5.085	0.000	1.370	1.214	1.547	0.136	1.146	1.099	1.194
HRV Group	−0.775	0.088	−8.808	0.000	0.461	0.330	0.643	−0.954	0.385	0.351	0.422
Maternal Type * HRV Group	−1.171	0.062	−18.893	0.000	0.310	0.158	0.608	−1.350	0.259	0.249	0.270
Reference	0.145	0.042	3.435	0.001	1.157	1.064	1.257	0.000	1.000	1.000	1.000
Maternal Type	0.345	0.061	5.614	0.000	1.411	1.251	1.592	0.200	1.221	1.177	1.268
PVI Group	0.095	0.061	1.541	0.124	1.099	0.975	1.240	−0.050	0.951	0.916	0.987
Maternal Type * PVI Group	0.061	0.087	0.702	0.483	1.063	0.897	1.260	−0.084	0.920	0.841	1.004
Reference	0.188	0.045	4.139	0.000	1.206	1.104	1.318	0.000	1.000	1.000	1.000
Maternal Type	0.470	0.061	7.651	0.000	1.601	1.419	1.806	0.282	1.326	1.285	1.368
SBP Group	0.005	0.061	0.089	0.929	1.005	0.891	1.134	−0.183	0.833	0.807	0.859
Maternal Type * SBP Group	−0.195	0.087	−2.238	0.026	0.823	0.694	0.976	−0.383	0.682	0.628	0.740
Reference	0.156	0.043	3.643	0.000	1.169	1.075	1.271	0.000	1.000	1.000	1.000
Maternal Type	0.369	0.062	5.974	0.000	1.446	1.281	1.632	0.213	1.237	1.192	1.284
Age Group	0.072	0.062	1.162	0.246	1.074	0.952	1.213	−0.084	0.920	0.886	0.954
Maternal Type * Age Group	0.018	0.087	0.208	0.835	1.018	0.858	1.208	−0.138	0.871	0.799	0.950
Reference	0.204	0.032	6.298	0.000	1.227	1.151	1.307	0.000	1.000	1.000	1.000
Maternal Type	0.404	0.046	8.790	0.000	1.498	1.369	1.640	0.200	1.221	1.193	1.250
Puncture siteL4 L5	−0.121	0.096	−1.261	0.208	0.886	0.734	1.069	−0.325	0.723	0.598	0.871
Maternal Type * Puncture siteL4 L5	−0.180	0.133	−1.349	0.178	0.835	0.643	1.085	−0.384	0.681	0.487	0.948
Reference	0.165	0.043	3.871	0.000	1.180	1.085	1.282	0.000	1.000	1.000	1.000
Maternal Type	0.330	0.062	5.363	0.000	1.391	1.233	1.569	0.165	1.179	1.142	1.217
Weight gain during pregnancy Group	0.414	0.062	6.676	0.000	1.514	1.189	1.934	0.249	1.283	1.246	1.320
Maternal Type * Weight gain during pregnancy Group	0.459	0.087	5.276	0.000	1.583	1.303	1.927	0.294	1.341	1.255	1.434

## 4 Discussion

Different anesthesia methods cause significant differences in physiological responses. Relevant studies have shown that spinal anesthesia, epidural anesthesia, and general anesthesia vary in their hemodynamic regulation (1, 12); meanwhile, different analgesia techniques, such as non-neuraxial analgesia, may also indirectly affect hemodynamics (13, 14). This study aimed to investigate the effect of maternal type (primiparas vs. multiparas) on the occurrence of hypotension after spinal anesthesia and analyzed the association between multiple factors and hypotension. We constructed univariate and multivariate regression models to evaluate the independent effect of maternal type on the risk of hypotension. The results showed that primiparas had a significantly higher risk of hypotension after spinal anesthesia compared to multiparas, and maternal type interacted with heart rate variability (HRV), systolic blood pressure (SBP), and gestational weight gain.

Firstly, regarding the impact of maternal type on the incidence of hypotension, primiparous women exhibit a significantly higher risk compared to multiparous women. This may be due to poorer blood volume and vascular reactivity in primiparas, as physiological changes during pregnancy and childbirth often render them more sensitive to spinal anesthesia (15). Our findings align with the observed higher PVI and heart rate in primiparous women, suggesting heightened sympathetic nervous system activity, which may contribute to an increased risk of hypotension following anesthesia (16). Furthermore, inclusion of maternal type in the multivariate model significantly enhanced its association with hypotension, highlighting maternal type as an important contributing factor. These results emphasize the need for individualized anesthesia plans and monitoring strategies in clinical practice to effectively prevent hypotension after spinal anesthesia, tailored to the specific characteristics of primiparous and multiparous women.

Our study found that lower perfusion index (PI), lower heart rate variability (HRV), higher pleth variability index (PVI), older maternal age, and greater gestational weight gain are all associated with an increased risk of hypotension. PI and PVI reflect blood perfusion and hemodynamic fluctuations (17, 18). Lower perfusion and greater variability often indicate unstable blood flow, which predisposes patients to hypotension (19). HRV represents the autonomic nervous system’s regulatory capacity; a reduced HRV typically suggests impaired autonomic function, resulting in an insufficient physiological response to hemodynamic changes after anesthesia and thereby increasing the likelihood of hypotension (20, 21). Excessive gestational weight gain can lead to a significant increase in blood volume and cardiac output, placing greater burden on the heart and potentially impairing the autonomic nervous system's ability to regulate blood pressure. This increased physiological load makes it more difficult for the body to maintain hemodynamic stability after the induction of anesthesia, thereby increasing the risk of hypotension (22). Conversely, higher baseline systolic blood pressure and selection of the L4–L5 puncture site were associated with a decreased risk of hypotension. Elevated baseline systolic pressure may reflect a stronger vasoconstrictive response, better enabling the body to counteract the blood pressure drop induced by spinal anesthesia (23, 24). Choosing the L4–L5 interspace for spinal anesthesia may be related to local anatomical considerations. Since the spinal cord terminates at the L1–L2 level, puncturing at the L4–L5 level ensures that the needle does not enter the spinal cord but rather affects the cauda equina or the subarachnoid space. Compared to the L2–L3 or L3–L4 levels, this lower puncture site allows for better control of the anesthetic spread, thereby reducing the risk of an excessively high block and excessive sympathetic blockade, which may help decrease the incidence of hypotension (25).

A key strength of this study lies in the interaction analysis, which revealed that baseline heart rate variability (HRV), baseline systolic blood pressure (SBP), and gestational weight gain are more strongly associated with spinal anesthesia-induced hypotension in primiparous women than in multiparous women. This disparity may stem from differences in autonomic nervous system regulation between the two groups during pregnancy. HRV, an indicator of sympathetic-parasympathetic balance, appears to be more sensitive in primiparas, who experience more pronounced physiological changes, thereby making HRV a stronger marker of hypotension risk. Similarly, baseline systolic blood pressure (SBP) is more strongly associated with spinal anesthesia-induced hypotension in primiparous women, possibly because they are more susceptible to postpartum blood pressure fluctuations, whereas multiparous women tend to have more stable hemodynamics. Gestational weight gain, which reflects cardiovascular load and hemodynamic shifts, also plays a more prominent role in primiparas, potentially due to less developed vascular adaptability compared to multiparas, who may have enhanced vascular responsiveness from prior pregnancies.

This study, through interaction analysis, can help anesthesiologists more accurately identify high-risk patients. Anesthesia providers can adjust anesthesia plans and monitoring strategies according to different types of parturients, thereby effectively preventing and promptly managing hypotension and improving anesthesia safety. Moreover, in primiparas, greater attention should be paid to baseline HRV, baseline SBP, and gestational weight gain. Dynamic monitoring and early intervention targeting these key indicators can more effectively prevent the occurrence of hypotension, ensure hemodynamic stability of the parturient, and further improve maternal and neonatal perinatal safety. Overall, this study provides a scientific basis for individualized anesthesia management and promotes a more precise and detailed approach to risk assessment in cesarean section anesthesia.

Currently, there is limited research exploring whether the association of spinal anesthesia-related hypotension indicators (such as PVI, HR, and HRV) differs between primiparous and multiparous women. Traditional hypotension prediction models are often based on single physiological indicators (such as SBP and HR), but they tend to overlook potential differences in these indicators across different maternal types. In this study, we incorporated interaction analyses involving maternal type, providing a more refined assessment perspective. This approach revealed how various physiological factors function differently in diverse populations, thus addressing some limitations of traditional analytical methods and offering more clinically valuable data support. However, this study has some limitations. This study was conducted at a single center with a relatively limited sample size, which may introduce regional bias. Moreover, all participants were Chinese parturients, and the findings may be influenced by the specific ethnic and regional characteristics of this population. In regions with different ethnicities, genetic backgrounds, and clinical practices, the risk factors for hypotension following spinal anesthesia may differ. Although we have included multiple influencing factors, confounding factors have not been completely excluded. For example, individual differences such as lifestyle habits and underlying medical conditions may affect hemodynamic responses. In addition, variations in anesthesia procedures (such as drug dosage and administration speed) and perioperative management strategies may also interfere with the incidence of hypotension. This study did not use standardized scoring scales like the Bromage or Hollmen scores for detailed assessment of motor and sensory block quality, which may limit the in-depth analysis of the relationship between the extent of anesthesia and the occurrence of hypotension. Future studies are recommended to include these scoring scales to improve the accuracy of block quality evaluation. Therefore, future studies should include multi-center, large-sample cohorts encompassing diverse ethnic populations to further validate the generalizability and applicability of our findings.

Previous studies have shown that pregnant women with systemic conditions such as COVID-19 infection often experience varying degrees of cardiopulmonary impairment. This is particularly evident in those with comorbidities such as obesity or hypertension, which may affect their tolerance to anesthesia and hemodynamic stability. During spinal anesthesia—where sympathetic tone is already physiologically reduced—autonomic dysfunction caused by COVID-19 may further exacerbate the incidence and severity of post-anesthesia hypotension (26). Against this backdrop, optimizing anesthesia management strategies becomes especially important. Existing research has indicated that adjuvant anesthetic agents such as sufentanil or dexmedetomidine can enhance the quality of anesthesia and reduce side effects by modulating the depth and duration of sympathetic blockade (27). These agents thus offer potential approaches for mitigating the risk of hypotension. In future clinical practice, particularly in pregnant women with high-risk underlying conditions, the careful selection of adjuvants and individualized adjustment of anesthesia protocols may play a critical role in preventing spinal anesthesia-induced hypotension.

## 5 Conclusion

This study demonstrates that maternal type significantly influences the occurrence of hypotension following spinal anesthesia during cesarean section, with primiparous women facing a higher risk compared to multiparous women. Furthermore, the interactions between maternal type and factors such as heart rate variability (HRV), systolic blood pressure (SBP), and gestational weight gain provide new insights for clinical interventions. These findings support the development of individualized anesthesia strategies to more effectively prevent hypotension. However, several limitations should be acknowledged: this was a single-center study with a relatively small sample size, which may limit the generalizability of the results. Additionally, as all participants were Chinese parturients, ethnic or regional differences may affect the applicability of the findings. Future studies should involve larger, multi-center cohorts including diverse ethnic populations to further validate and extend these findings.

## Data Availability

The original contributions presented in the study are included in the article/Supplementary Material, further inquiries can be directed to the corresponding author.
